# Exploring the safety and efficacy of phytomedicine *Petiveria alliacea* extract (Esperanza) in patients with metastatic gastrointestinal tumors and acute leukemias: study protocol for a phase Ib/randomized double blind phase II trial (PA001)

**DOI:** 10.1186/s12906-023-04109-2

**Published:** 2023-08-10

**Authors:** Ricardo Ballesteros-Ramírez, Paola Pinilla, Jesús Sanchéz, Mónica Arévalo, Elio Sanchez, Pablo Aschner, Claudia Urueña, Susana Fiorentino

**Affiliations:** 1 Grupo de Inmunobiología Y Biología Celular, Facultad de Ciencias, Pontificia Universidad Javeriana, Carrera 7 No. 43-82, Edificio Félix Restrepo, Lab 101. , C.P.110211 Bogotá, Colombia; 2 Centro Javeriano de Oncología, Hospital Universitario San Ignacio, Bogotá, Colombia; 3 Oficina de Investigaciones, Hospital Universitario San Ignacio, Bogotá, Colombia

**Keywords:** *Petiveria alliacea*, Herbal Drugs, Safety, Efficacy, Tumoral metabolism, Quality of Life

## Abstract

**Background:**

The energy metabolism of drug-resistant tumor cells can provide a survival advantage during therapy, and treatment itself may influence metabolic reprogramming. *Petiveria alliacea* (Traditional name: Anamu*)* could inhibit glycolysis and OXPHOX modulating tumor metabolism, making it a potential treatment for tumors with altered metabolism. This clinical study aims to evaluate the safety and efficacy of a standardized Anamu phytomedicine called Esperanza in treating gastric tumors and acute leukemias.

**Methods:**

This is a prospective, open label, phase I/ randomized, double-blind single-center phase II study designed to evaluate the safety and efficacy of Esperanza extract in patients with metastatic gastrointestinal tumors and acute leukemias. In stage 1, the study will determine the MTD and assess safety. In stage 2, safety at the MTD will be evaluated, and the efficacy of Esperanza extract will be explored in both metastatic gastric tumors and acute leukemias. Quality of life improvement will be the primary outcome in the gastric tumor group, while different efficacy outcomes will be assessed in the acute leukemia group. A placebo group will be used for comparison in the gastric tumor group, and a historical control group will be used in the acute leukemia arm.

**Discussion:**

This clinical trial aims to evaluate the safety profile of the Esperanza extract in patients with metastatic gastrointestinal tumors and acute leukemias, while exploring its potential efficacy in conjunction with standard treatment for these pathologies.

**Trial registration:**

This trial was registered in the US National Library of Medicine with identifier NCT05587088. Registered October 19^th^, 2022.

**Supplementary Information:**

The online version contains supplementary material available at 10.1186/s12906-023-04109-2.

## Administrative information

**Table:** 

Title {1}	Exploring the Safety and Efficacy of Phytomedicine *Petiveria alliacea* Extract (Esperanza) in Patients with Metastatic Gastrointestinal Tumors and Acute Leukemias: Study protocol for a phase Ib/randomized double blind phase II trial (PA001)]
Trial registration {2a and 2b}	US National Library of Medicine: NCT05587088. Registered October 19^th^, 2022
Protocol version {3}	Protocol PA001 Evaluation of the safety of the extract Esperanza (*Petiveria alliacea*) in patients with metastatic gastrointestinal tumors and acute leukemias and exploration of its efficacy, Version 1.1 of March 3, 2023, replaces version 1.0 of August 24, 2022
Funding {4}	Funding is provided by the Minister of Science Technology and Innovation (792–2017 2nd Call Scientific Ecosystem). Project: “Generation of therapeutic alternatives in cancer from plants through research and translational development, articulated in environmentally and economically sustainable value systems”), World Bank and Vicerrectoría de Investigaciones, Pontificia Universidad Javeriana, Bogotá, Colombia (contract no. FP44842-221–2018), MINCIENCIAS, Ministerio de Educación Nacional, Ministerio de Industria, Comercio y Turismo and ICETEX. Contract for access to genetic resources 212 of July 19, 2018 signed between the Pontificia Universidad Javeriana and the Ministry of the Environment of Colombia
Author details {5a}	Ricardo Ballesteros-Ramírez^1^, Paola Pinilla^2^, Jesús Sanchéz^2^, Mónica Arévalo^2^, Elio Sanchez^2^, Pablo Aschner^3^, Claudia Urueña^1^, Susana Fiorentino^1*^ ^1^Grupo de Inmunobiología y Biología Celular, Facultad de Ciencias, Pontificia Universidad Javeriana. Bogotá, Colombia ^2^Centro Javeriano de Oncología, Hospital Universitario San Ignacio. Bogotá, Colombia ^3^Oficina de Investigaciones, Hospital Universitario San Ignacio. Bogotá, Colombia Carrera 7 No. 43–82, Edificio Félix Restrepo, Lab 101. Bogotá C.P.110211, Colombia *Corresponding author: Prof. Dr. Susana Fiorentino susana.fiorentino@javeriana.edu.co
Name and contact information for the trial sponsor {5b}	Prof. Dr. Susana Fiorentino Director of the Immunobiology and Cell Biology Group Research Unit in Biomedical Sciences Science Faculty Pontificia Universidad Javeriana Email: susana.fiorentino@javeriana.edu.co
Role of sponsor {5c}	This clinical trial is part of the GAT Program – Scientific Ecosystem, which is headed by Prof. Dr. Susana Fiorentino. The ecosystem is focused on developing therapeutic options from plant-based sources that comply with FDA regulatory standards. The trial specifically aims to advance the development of Anamu phytomedicine and is spearheaded by Prof. Dr. Susana Fiorentino. The clinical study is being conducted exclusively at the Centro Javeriano de Oncología at the Hospital Universitario San Ignacio

## Introduction

### Background and rationale {6a}

The development of drug-resistant tumor cells is a major challenge in cancer treatment. Tumor cells with altered energy metabolism may have an advantage during adaptation to therapy, and the type of therapy can influence metabolic reprogramming [1, 2]. This means that attacking a single molecular target or metabolic pathway may favor the selection of chemotherapy-resistant clones [3]. Additionally, traditional cancer treatments may not necessarily reduce metastasis. Metastatic colonization requires an increase in adenosine triphosphate (ATP) production and a metabolic "rewiring" that reduces the production of reactive oxygen species (ROS) and redirects the toxic radical scavenging capacity to the mitochondria, making antioxidant defenses important for favoring metastasis [4].

Considering the significant intratumoral metabolic heterogeneity and plasticity observed in tumors, it is improbable that single agents will prove effective as a cancer treatment. Instead, combined therapies that simultaneously target two or more metabolic pathways can effectively impede relapse and the emergence of resistance. For instance, the complete eradication of tumor stem cells (TMCs) within the tumor can be achieved by dual inhibition of the primary metabolic pathway and its key escape mechanism. Such results have been documented in studies combining metformin with the bromodomain and extra-terminal (BET) inhibitor, JQ-1, in pancreatic cancer [5], as well as through phosphatidylinositol-3-kinase (PI3K) inhibition in ovarian cancer [6]. These combinations block oxidative phosphorylation (OXPHOS) and indirectly inhibit glycolysis simultaneously. Current understanding underscores the significance of meticulously designed therapies that target metabolically diverse populations and account for the tumor microenvironment. These considerations are paramount in developing more effective treatment strategies focused on metabolism [7].

Traditional Chinese medicine (TCM) has been used for thousands of years and has become a treatment option in many cancer centers worldwide [8]. TCM and some traditionally used plants interfere with cancer metabolism through various pathways and targets, including glycolysis and fatty acid synthesis [9]. The altered metabolism also negatively affects the activation of the immune response, so antitumor treatment focused on metabolic dysfunction may allow for better activity of the immune response and better response to biological therapies with antibodies against immune response checkpoints.

In Colombia, there is a significant collection of medicinal plants used by indigenous communities, some of which also coexist in other regions of the world. *P.alliacea* (*Petiveria alliacea*) is one such plant, which has shown enormous therapeutic potential in various ethnopharmacological studies [10–12]. Methanolic extracts of *P.alliacea* have exhibited cytotoxic activity on various cancer cell lines, including hepatocellular carcinoma cells, melanoma cells, leukemia cells, breast cancer cells, and colon cancer cells [13]. The whole plant decoction has been shown to be an immunostimulant, activating murine splenocytes and stimulating the production of interferon and interleukins 4 and 2, as well as increasing the activity of natural killer cells [14]. Hexanolic extract of *P.alliacea* has been shown to increase the phagocytic index of human granulocytes, and hydroalcoholic extracts have demonstrated immunomodulatory activity by increasing phagocytosis and activating NK cells with increased IL-2 and IFNg production. In contrast, anti-inflammatory activities have been seen in other experimental models, where the extract decreased the formation of granulomas and dermatitis in rodents previously treated with proinflammatory agents [15, 16].

In our group, we have identified several activities of the *P.alliacea* extracts that affect tumor metabolism. Notably, the extract can modulate various proteins associated with cell metabolism, including peroxiredoxin 6, glucose phosphate isomerase, ACLY variant protein, phosphoglycerate dehydrogenase, pyruvate kinase, enolase 1, fatty acid synthase, lactate dehydrogenase A, phosphoglycerate kinase (PGK), ATP synthase-H transporter, mitochondrial F1 complex, glyceraldehyde-3-phosphate dehydrogenase, glucosidase II, prostaglandin E synthase 3 (cytosolic), and dihydropyrimidinase variant type 2. Furthermore, chaperone proteins such as Hsp70, Hsp60, tumor rejection antigen (gp96), Heat Shock Proteins 90 (Hsp90), and Hsp90alpha were downregulated after extract treatment. These proteins are crucial for cell survival and protection against stressful stimuli, highlighting the extract's potential as an effective therapeutic agent against cancer [17]. The *P.alliacea* extract has demonstrated specific cytotoxic activity against various breast cancer cell lines, while sparing fibroblasts and normal epithelial cells. It has also been found to reduce glycolytic flux early, leading to a decrease in mitochondrial β-F1-ATPase and a marked reduction in mitochondrial ATP levels. These metabolic alterations correlate with reduced spheroid formation and proliferation capacity of 4T1 cells in vitro, as well as decreased tumor size in TS/A murine breast cancer tumor cell transplants [18, 19]. Our group has also shown that the aqueous fraction of *P. alliacea* induces partial maturation of human dendritic cells and the production of pro- and anti-inflammatory cytokines, while the organic fraction does not have immunomodulatory activity. These findings suggest that the antitumor effects of the aqueous extract of *P. alliacea* occur through various mechanisms, including the regulation of tumor metabolism, reduction of inflammation, and modulation of the immune response [20]. Additionally, the Anamu extract, which does not exhibit significant antioxidant activity, induces the death of blasts from patients with Acute Lymphoid Leukemia and a greater proportion of Acute Myeloid Leukemia, especially those from relapsed patients, as demonstrated in primary leukemia cell analysis [21].

Despite the significant amount of research done on *P. alliacea,* more controlled clinical studies are needed to validate its therapeutic potential in cancer.

### Objectives {7}

The main goal of phase I is to assess the safety, toxicity, and maximum tolerated dose (MTD) of the Esperanza extract in patients diagnosed with metastatic gastric tumors and acute leukemias. In phase II, the focus shifts to evaluating the safety of the extract and examining its impact on improving patients' quality of life using the QLQ-C30 scale for gastric tumors. Additionally, in the context of acute leukemias during phase II, the emphasis remains on determining the safety of the extract and investigating its potential to enhance the rate of remission during induction therapy and/or facilitate hematological recovery.

In addition to the primary objectives, there are important secondary objectives for gastric tumors. One of these is to evaluate the influence of the Esperanza extract on progression-free survival and clinical response, which will be assessed using the RECIST 1.1 criteria. For acute leukemias, the assessment will focus on event-free survival, defined as the duration from enrolment to disease progression, including local or distant recurrence, second primary malignancy, primary malignancy-related death, or death from any cause, whichever comes first. Furthermore, a thorough evaluation of the impact on patients' quality of life will also be conducted.

### Trial design {8}

**Fig. 1:**
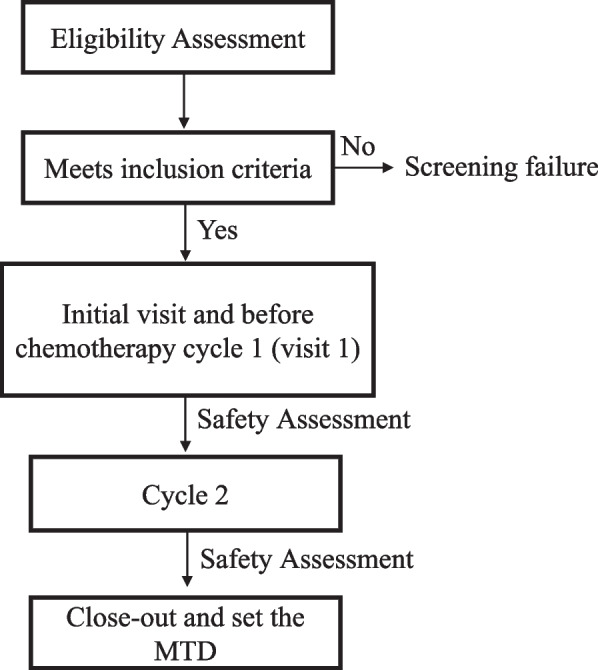
Flow diagram for phase I in gastrointestinal tumors and acute leukemias

**Fig. 2:**
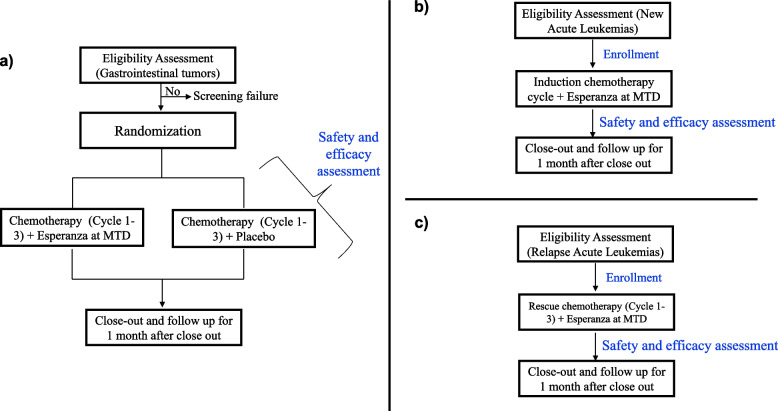
Comprehensive Flow Diagram for Phase II PA001 Trial **a**) Gastrointestinal Tumors, **b**) New diagnosis Acute Leukemias, and **c**) Relapsed Acute Leukemias

This is a comprehensive phase I/II clinical study designed to assess both the safety and efficacy of the Esperanza extract in treating gastric tumors and acute leukemias. The study consists of two phases: Phase I is an open-label study that involves dose escalation to determine the MTD of the extract (Fig. 1). In Phase II, specifically for gastric tumors, a double-blind, randomized, placebo-controlled approach will be employed. Participants will be divided into two groups, with both groups receiving standard chemotherapy in combination with either the Esperanza phytomedicine or a placebo (Fig. 2a).

Conversely, in the acute leukemia group, the MTD will be administered to patients during the induction cycle. The results from these acute leukemia cohorts will be compared to a historical cohort at the hospital, allowing for further analysis and evaluation (Fig. 2b). In relapse acute leukemia patients, the MTD will be administered to patients during the rescue cycles 1 to 3, the results will be compared to a historical cohort at the hospital (Fig. 2c).

## Methods: Participants, interventions, and outcomes

### Study setting {9}

This study aims to assess the safety and investigate the efficacy of the Esperanza extract in treating gastric tumors and acute leukemias. The research will be exclusively conducted at the Hospital Universitario San Ignacio. During Phase I, the safety profile of the extract will be evaluated when used in combination with standard chemotherapy for each specific tumor type. In Phase II, the focus will shift to exploring the extract's efficacy while continuing to assess its safety.

### Eligibility criteria {10}

#### Inclusion criteria

##### Gastrointestinal tumors:

1. Patients aged 18 years or older, with no upper age limit.
2. Histological diagnosis of gastrointestinal cancer, including stomach, colon, bile ducts, and pancreas.
3. Measurable disease confirmed by diagnostic tests and planned to receive chemotherapy as the primary treatment.
4. Presence of at least one discrete metastatic site identified by CT scan of the chest and/or abdomen and/or bone scintigraphy.
5. ECOG performance status of 0 to 2, with a life expectancy greater than 2 months.
6. Ability to swallow and retain oral medication, and absence of uncontrolled emesis or persistent diarrhea.
7. Adequate renal, hepatic, and cardiac function defined as follows:
  Renal Function: Glomerular filtration rates greater than 30 mL/min, determined by blood chemistry (CKD-EPI calculation) and/or urinary creatinine clearance and/or Glomerular filtration scintigraphy.
  Liver Function: Alkaline phosphatase, ALT, and AST should not exceed 5 times the reference value according to laboratory technique. Bilirubin should not exceed 3 times the reference value.
  Cardiac Function: Echocardiogram and/or electrocardiogram results should indicate no untreated or decompensated heart failure, with an ejection fraction greater than or equal to 50%. Additionally, any value lower than the mentioned threshold, posing a risk to the patient, should be assessed by the investigator.
8. Female patients of childbearing age who are not using safe non-hormonal contraceptive methods must undergo a negative pregnancy test prior to screening.
9. Fertile female subjects (not postmenopausal for at least 12 months or surgically sterile) and their male partners must use one of the following contraceptive methods from study entry, throughout the study, and for at least 6 months after using Esperanza extract (unknown effects on the developing fetus):
  Complete abstinence from sexual intercourse, beginning at least one complete menstrual cycle before study drug administration (note: sexual abstinence as a contraceptive method should be limited to cases where it is already an established choice of the patient's pre-existing lifestyle).
  Vasectomy in the partner of a female subject.
  Intrauterine device (IUD).
  Double barrier method (condom, contraceptive sponge, diaphragm, or vaginal ring with spermicidal jelly or cream).
10. Willingness to complete the study and comply with follow-up interventions.

##### Acute Leukemias

1. Patients aged 18 years or older, with no upper age limit.
2. Patients diagnosed with acute myeloid leukemia (AML) or acute lymphoblastic leukemia (ALL) and eligible for chemotherapy treatment, meeting the following requirements:
  Newly diagnosed patients as per WHO 2022 criteria, determined by hematic picture and/or myelogram and/or flow cytometry.
  Patients who have failed initial therapy or during consolidation treatment, characterized by either primary refractory disease (induction treatment failure in the first treatment cycle) or evidence of relapse (after achieving complete remission, during consolidation or maintenance therapy).
3. Ability to swallow and retain oral medication, without uncontrolled emesis or persistent diarrhea. Adequate renal, hematological, hepatic, and cardiac function defined as follows:
  Ejection fraction greater than or equal to 50%.
  Creatinine clearance greater than 45 ml/min, calculated by the Janowitz formula or measured with a 24-h urine collection.
  Total bilirubin greater than 1.5 times the laboratory reference value or greater than 3 times the reference value if attributed to neoplastic disease.
  Transaminases greater than or equal to 3.0 times the laboratory reference value.
4. Female patients of childbearing age without safe non-hormonal contraceptive methods must undergo a negative pregnancy test before screening.
5. Fertile female subjects (not postmenopausal for at least 12 months or surgically sterile) and their male partners must use at least one of the following contraceptive methods from study entry, throughout the study, and for at least 6 months after using Esperanza extract (unknown effects on the developing fetus):
  Complete abstinence from sexual intercourse, beginning at least one complete menstrual cycle before study drug administration (note: sexual abstinence as a contraceptive method should be limited to cases where it is already an established choice of the patient's pre-existing lifestyle).
  Vasectomy in the partner of a female subject.
  Intrauterine device (IUD).
  Double barrier method (condom, contraceptive sponge, diaphragm, or vaginal ring with spermicidal jelly or cream).
6. Willingness to complete the study and comply with follow-up interventions.

#### Exclusion criteria

Subjects meeting any of the following conditions are ineligible for participation in this study:

1. Individuals who have received treatment as part of any other therapeutic clinical protocol within the 30 days preceding study entry or during the course of the study.
2. Patients currently receiving other investigational agents.
3. Female subjects who are pregnant or lactating. Confirmation of non-pregnancy must be established through a negative result on a serum or urine pregnancy test conducted during the screening process. Postmenopausal or surgically sterilized women are exempt from undergoing pregnancy tests.
4. Individuals with serious concomitant morbidities that are active as determined by the investigator, including:
  Untreated or uncontrolled arterial hypertension, or values consistently above 140/90 mmHg despite treatment.
  Untreated or decompensated heart failure according to the Stevenson scale.
  Coronary artery disease without intervention, confirmed by catheterization, or symptomatic angina.
  Clinically or electromyographically documented neuropathy.
  Liver cirrhosis/hepatic dysfunction without treatment or values that meet the exclusion criteria.
  Chronic kidney disease with a glomerular filtration rate (GFR) less than 30 ml/min.
  Untreated or decompensated autoimmune diseases.
  Diabetes without treatment or with HbA1c levels greater than 8% despite treatment.
  Dyslipidemia without treatment or with triglyceride values greater than 300 or LDL levels greater than 200 despite treatment.
  Synchronous or metachronous active tumors (hematological or solid) within the 2-year period prior to study entry.
  Any other concomitant disease, not mentioned above, that, in the investigator's opinion, poses a risk to the patient and is not a suitable substitute.
5. Subjects with a confirmed diagnosis of HIV prior to enrollment or a positive HIV diagnosis at the time of screening.
6. Patients who have undergone solid organ transplants.
7. Any other condition, as determined by the principal investigator, that renders the subject ineligible to participate in this study.

### Who will take informed consent? {26a}

The informed consent process will be conducted by the principal investigator or a designated representative. They will thoroughly explain the study's objectives, procedures, and the potential benefits and risks of participation to each prospective patient. Prior to undergoing any study-related procedures or discontinuing any prohibited medication, every patient or their legally acceptable representative must sign and date the informed consent form approved by the Institutional Review Board (IRB).

### Additional consent provisions for collection and use of participant data and biological specimens {26b}

N/a: The collection of all data and biological samples for this study is an integral part of the protocol activities.

## Interventions

### Explanation for the choice of comparators {6b}

In Phase Ib, no comparator will be used as the primary objective is to determine the maximum tolerated dose (MDT) in gastric tumors and acute leukemias. In Phase II, a placebo group will be included for gastric tumors, while both groups will receive the standard chemotherapy for their respective underlying diseases. However, for acute leukemias, a comparison will be made with a cohort of historical records from the hospital where the relevant clinical outcomes have already been established, considering the causal factors.

### Intervention description {11a}

#### Phase I treatment:

Patients who meet the inclusion criteria and have metastatic gastrointestinal tumors (colon, pancreas, stomach, and bile ducts) or newly diagnosed acute leukemias will initiate treatment with 1000 mg of Esperanza extract daily, divided into two doses of 500 mg, for a duration of 4 weeks. In the event of toxicity, the dose level will be adjusted accordingly. If toxicity occurs, the next dose level will be reduced to 500 mg using the same dosing schedule, and if there is no toxicity after 4 weeks, the dose will be increased to 2000 mg. This dose adjustment will be conducted with the same group of patients, both for escalation and de-escalation, who have completed the previous dose level, ensuring a total treatment duration of 8 weeks. The administration of the phytomedicine Esperanza will continue throughout the intervention period, without interruption, for the entire 8-week treatment duration, unless there is clinically significant toxicity that meets the investigator's criteria for discontinuing the intervention. Table 1 provides a comprehensive list of the activities to be carried out during the patients' intervention.

**Table 1:** Schedule of activities for phase ib to evaluate safety and MTD in gastrointestinal tumors and acute leukemias

Schedule of activities				
	**Enrollment**	**Initial Visit and chemotherapy cycle 1**	**Cycle 2**	**Close-out**
**Time point**	**Week 1**	**Week 2**	**Week 6**	**Week 7**
Enrolment				
Screening and Informed Consent	X			
Physical Examen		X	X	X
Clinical Record		X	X	X
HIV Test and Pregnancy test	X			
**Interventions**				
Medication dispensing		**X**	**X**	**X**
Follow-up visit		**X**	**X**	**X**
**Safety Assessment**				
Hemogram		X	X	X
Transaminase AST		X	X	X
Transaminase ALT		X	X	X
Alkaline Phosphatase		X	X	X
Total Bilirubin		X	X	X
Triglycerides		X	X	X
Total Cholesterol		X	X	X
Blood Urea Nitrogen (BUN)		X	X	X
Creatinine		X	X	X
Urinalysis		X	X	X
Uric Acid		X	X	X
LDH (Lactate Dehydrogenase)		X	X	X
Lactic Acid		X	X	X
Ferritin		X	X	X
Albumin		X	X	X
Iron		X	X	X
Electrocardiogram		X	X	X
Echocardiogram				

#### Phase II treatment:

For patients with metastatic gastrointestinal tumors (colon, pancreas, stomach, and bile ducts), a total of 30 new patients will be enrolled and divided into two groups: an intervention group and a placebo group, each consisting of 15 patients. The intervention group will receive continuous treatment with the Esperanza extract at the determined maximum tolerated dose (MTD) for three treatment cycles, equivalent to approximately 12 weeks. The placebo group will receive a placebo in addition to standard chemotherapy treatment for their underlying disease. Safety evaluations will be conducted at the end of each treatment cycle, and the efficacy evaluation will be carried out after completion of the three treatment cycles. Table 2 provides a comprehensive list of the activities to be carried out during the patients' intervention.

**Table 2:** Activity schedule for phase II clinical trial in gastrointestinal tumors

Schedule of activities					
**Gastrointestinal Tumors**					
	**Enrollment**	**Initial Visit and chemotherapy cycle 1**	**Cycle 2**	**Cycle 3**	**Close-out**
**Time point**	**Week 1**	**Week 2**	**Week 6**	**Week 10**	**Week 12 -14**
Enrolment					
Screening and Informed Consent	X				
Physical Examen		X	X	X	X
Clinical Record		X	X	X	X
HIV Test and Pregnancy test	X				
**Interventions**					
Medication dispensing		**X**	**X**	**X**	**X**
Follow-up visit		**X**	**X**	**X**	**X**
**Safety Assessment**					
Hemogram		X			X
Transaminase AST		X			X
Transaminase ALT		X			X
Alkaline Phosphatase		X			X
Total Bilirubin		X			X
Triglycerides		X			X
Total Cholesterol		X			X
Blood Urea Nitrogen (BUN)		X			X
Creatinine		X			X
Urinalysis		X			X
Uric Acid		X			X
LDH (Lactate Dehydrogenase)		X			X
Lactic Acid		X			X
Ferritin		X			X
Albumin		X			X
Iron		X			X
Electrocardiogram		X			X
Echocardiogram		X			X
**Efficacy Assessment**					
RECIST		X			X
Tumor Antigens		X			X
CA199		X			X
ACE		X			X
Metabolome		X		X	X
Microbiome Analysis		X			X
Proinflammatory Cytokines		X		X	X
Circulating DNA		X		X	X
Ketone Bodies		X		X	X
Tumor Infiltration		X			X
QLQ C-30 Quality of Life Surveys		X	X	X	X
Progression-Free Survival		X			X

For newly diagnosed acute leukemias, a total of 28 new patients will be recruited, and an additional six patients will be recruited for the relapsed leukemias group. Both groups will receive continuous treatment with the Esperanza extract at the determined MTD for four weeks. Standard chemotherapy treatment for their underlying disease will be administered to both groups. At the end of the treatment cycle, safety and efficacy evaluations will be conducted. Table 3 provides a comprehensive list of the activities to be carried out during the patients' intervention.

**Table 3:** Activity schedule for phase II clinical trial in acute leukemias

Schedule of activities				
**Acute leukemias**				
	**Enrollment**	**Initial Visit and chemotherapy cycle 1**	**Cycle 2**	**Close-out**^**a**^
**Time point**	**Week 1**	**Week 2**	**Week 6**	**Week 10**
Enrolment				
Screening and Informed Consent	X			
Physical Examen		X	X	X
Clinical Record		X	X	X
HIV Test and Pregnancy test	X			
**Interventions**				
Medication dispensing		**X**	**X**	**X**
Follow-up visit		**X**	**X**	**X**
**Safety Assessment**				
Hemogram		X		X
Transaminase AST		X		X
Transaminase ALT		X		X
Alkaline Phosphatase		X		X
Total Bilirubin		X		X
Triglycerides		X		X
Total Cholesterol		X		X
Blood Urea Nitrogen (BUN)		X		X
Creatinine		X		X
Urinalysis		X		X
Uric Acid		X		X
LDH (Lactate Dehydrogenase)		X		X
Lactic Acid		X		X
Ferritin		X		X
Albumin		X		X
Iron		X		X
Electrocardiogram		X		X
Echocardiogram		X		X
**Efficacy Assessment**				
Myelogram and flow cytometry		X		X
Tumor Antigens		X		X
Metabolome		X		X
Microbiome Analysis		X		X
Proinflammatory Cytokines		X		X
Circulating DNA		X		X
Ketone Bodies		X		X
Tumor Infiltration		X		X
QLQ C-30 Quality of Life Surveys		X	X	X
Progression-Free Survival		X		X

^a^For new diagnosis the intervention will be only in induction chemotherapy cycle with the close out visit in week 6

The Esperanza and Placebo capsules will be manufactured at Labfarve Laboratories in strict adherence to Good Manufacturing Practices (GMP) conditions. The Esperanza capsules contain 500 mg of *P. alliacea* extract, which is obtained from freshly crushed leaves infused with purified water. The extraction process has a DER (Drug extraction ratio) of 29–44:1, and the hexose content ranges between 3.0% and 5.0%. Each batch undergoes chemical profile assessment to ensure consistency. Quality controls are conducted in accordance with regulations set forth by the World Health Organization (WHO), the National Institute for Food and Drug Surveillance (INVIMA), and the U.S. Food and Drug Administration (FDA). These controls will be applied to both the investigational phytomedicine and the placebo, guaranteeing an identical physical appearance and a shelf life of six months from the date of manufacture.

### Criteria for discontinuing or modifying allocated interventions {11b}

Each subject retains the right to voluntarily withdraw from the study treatment at any given time. Moreover, the investigator holds the authority to suspend a subject from the study treatment if deemed necessary for various reasons, such as the occurrence of adverse events or non-adherence to the study protocol. Instances where a subject may be withdrawn from the study treatment include: withdrawal of consent by the subject or their legally acceptable representative, experiencing unacceptable toxicity (grade ≥ 3) with the combination of Esperanza and standard therapy according to NCI CTCAE version 5.0, as well as cases where the investigator determines the toxicity to be clinically intolerable. Additionally, suspension may occur if there is a clinically significant elevation that falls below grade 3 or 4, at the discretion of the investigator. Significant non-compliance with the protocol that could jeopardize the subject's safety or compromise data integrity also warrants withdrawal. The investigator may also make the decision to withdraw a subject if it is believed to be in the subject's best interest. Lastly, a positive pregnancy test will result in immediate withdrawal from the study treatment.

### Strategies to improve adherence to interventions {11c}

All participants are scheduled for regular follow-up visits, during which a medication count is conducted to assess adherence rates. In addition, participants can address any questions they may have, and the evaluation of adverse effects that occurred during the intervention is performed. Furthermore, a telephone follow-up is carried out weekly, ensuring that participants can directly contact the coordinator to seek clarification, resolve doubts, or report any abnormal clinical signs they may be experiencing.

### Relevant concomitant care permitted or prohibited during the trial {11d}

#### Metastatic gastrointestinal tumors:

Patients will receive standard therapy as determined by their oncologist, which may involve treatment regimens incorporating platinums, topoisomerase inhibitors, fluoropyrimidines, or antimetabolites. The specific doses will be determined by the patient's clinical oncologist in accordance with the institutional, national, and international guidelines adopted by the Hospital Universitario San Ignacio for metastatic gastrointestinal tumors. In addition to the chemotherapy protocol, antiemetic agents such as setrons and steroids, as well as fosaprepitant administered prior to infusion, may be included. Oral setrons every 12 h for approximately 5 days, proton pump inhibitors like omeprazole, and antidiarrheals such as loperamide (especially in the case of taxanes) may also be prescribed. Colony-stimulating factors such as Pegfilgastrim and folic acid analogues may be utilized.

#### Newly diagnosed and/or relapsed acute leukemias:

Patients will receive standard therapy as determined by their hematologist, which may involve treatment regimens utilizing anthracyclines, nitrogenous mustards, vinca alkaloids, antimetabolites, and topoisomerase inhibitors. The specific doses will be determined by the patient's clinical hematologist in accordance with the institutional, national, and international guidelines adopted by the Hospital Universitario San Ignacio for acute leukemias. Alongside the chemotherapy protocol, antiemetic agents such as setrons and steroids, as well as fosaprepitant administered prior to infusion, may be included. Oral setrons every 12 h for approximately 5 days, proton pump inhibitors like omeprazole, and antidiarrheals such as loperamide (especially in the case of taxanes) may also be prescribed. Colony-stimulating factors such as Pegfilgastrim/Filgastrim and folic acid analogues may be utilized.

### Provisions for post-trial care {30}

All participants will be monitored for a period of 1 month following the completion of their treatment. Additionally, after their final visit, participants will be informed that they have the option to seek clarification or report any new signs or symptoms through telephone or in-person consultations. Furthermore, patients will continue to receive regular clinical follow-up for their underlying disease, adhering to the established schemes and protocols at the Hospital.

### Outcomes {12}

The primary outcome of this study is to determine the safety, efficacy, and maximum tolerated dose (MTD) of the administration of the Esperanza extract in patients with metastatic gastrointestinal tumors as well as in patients with acute leukemias. Additionally, the study aims to assess the impact of the Esperanza extract on the quality of life of these patients using the QLQ-C30 scale. The evaluation period will be between 4 and 6 months, and the QLQ-C30 version 3 scale will be employed as the standardized method for assessing the patients' quality of life outcomes.

The secondary outcomes of this study are as follows:

- To determine the effect of treatment with the Esperanza extract in combination with chemotherapy on the clinical and pathological response. This evaluation will be conducted between 1 and 3 months using the Recist 1.1 (Response Evaluation Criteria In Solid Tumors) scale as the standardized evaluation method and with flow cytometry and myelogram in acute leukemias.
- To assess the impact of treatment with the Esperanza extract in combination with chemotherapy on the number and size of metastases. This assessment will also be carried out between 1 and 6 months, utilizing the Recist 1.1 for gastrointestinal tumors and cytometry and myelogram for acute leukemias.
- To determine progression-free survival and overall survival. The evaluation period for progression-free survival will be 3 months, and the Log Rank test will be used as the evaluation method. Progression-free survival will be measured from randomization to the occurrence of disease progression, local or distant recurrence, development of a second primary malignancy, or death from any cause, whichever occurs first. Patients who do not meet the evaluation time point will be censored and followed up until the end of treatment for survival curve preparation.

### Participant timeline {13}

#### Sample size {14}

For phase I, a sample size of 6 patients was determined for gastric tumors and acute leukemias. In case of presentation of any adverse event in the first in these patients, the dose will be decreased for the second cycle; in case of an adequate safety profile, the dose will be doubled for the second cycle.

The sample sizes for phase II were determined for both gastrointestinal tumors and hematologic tumors. For gastrointestinal tumors, a sample size of 30 patients was calculated, with 15 patients per group, considering a mean difference of at least 6 points in quality of life, a standard deviation of 5 and 6 points for each group, an alpha error of 0.05, and a beta error of 0.20. Additionally, a 10% allowance was added for possible study losses. For hematologic tumors, the sample size calculation was based on response criteria such as improvement, remission rate, and hematologic recovery. The ANOVA test was used, an alpha error of 0.05, a beta error of 0.20, and an effect size of 0.4. The resulting sample size was 28.

It should be noted that these sample sizes will be reassessed at the end of phase I for both gastric tumors and acute leukemia. The recalculated sample size for phase I/II is 36 for gastrointestinal tumors and 46 for hematologic tumors.

### Recruitment {15}

The recruitment of patients will start in July 2023 and conclude by February 2023 at the Centro Javeriano de Oncologia of the Hospital Universitario San Ignacio. As a renowned cancer treatment center in Colombia, the San Ignacio University Hospital offers comprehensive oncology programs, enabling all clinical staff involved in the study to identify and track potential candidates. Preselected patients will be contacted and provided with a detailed explanation of the study, along with the informed consent form. Subsequently, an enrollment visit will be conducted to ascertain the patient's decision regarding participation. Once enrolled, each patient will be assigned a dedicated coordinator responsible for organizing visits and follow-ups, ensuring the seamless execution of study activities. Patients will be informed of their right to withdraw from the study at any time without compromising their ongoing clinical treatment.

## Assignment of interventions: allocation

### Sequence generation {16a}

During the screening visit, all participants will be assigned a unique subject number in sequential order (e.g., 001, 002, 003, etc.), which will remain consistent throughout the study. The treatment chosen by the attending physician will be administered to all participants, without randomization in phase I.

In phase II, patients with gastrointestinal tumors will undergo randomization using a specially designed application within the eCRF in RedCap®. The randomization will be conducted using a simple non-stratified approach, predetermined, and loaded into the eCRF for the pharmacist, who will be unblinded. Randomization will not be employed for acute leukemias, as the comparison of outcomes will be conducted against a historical group using propensity score matching.

### Concealment mechanism {16b}

Randomization will be performed using the eCRF module in RedCap® that is preloaded specifically for this study prior to its initiation. This module allows for efficient and accurate random assignment of participants to their respective treatment groups.

### Implementation {16c}

Participants will be recruited by the clinical staff involved in the study, and the informed consent process will be conducted by the principal investigator or a designated delegate, following the established procedures at the San Ignacio University Hospital. Once participants have signed the consent form, if applicable, the pharmacist will assign them to either the placebo or intervention group for dispensing. The blinding of the study will be maintained until all participants have been assigned to their respective groups, and it will only be opened by designated personnel responsible for the analysis of the results at the conclusion of the study.

## Assignment of interventions: Blinding

### Who will be blinded {17a}

In Phase I, both the patients and the members of the research team are aware that the patients are receiving the research phytomedicine, Esperanza. However, in Phase II for gastric tumors, a double-blind approach will be implemented. This means that both the patients and the members of the research team, except for the pharmacist, will be unaware of whether the patients are receiving the placebo or the intervention. The pharmacist, being the only non-blind staff member, will have knowledge of the assigned treatments for dispensing purposes.

### Procedure for unblinding if needed {17b}

In the event of serious adverse events that, in the opinion of the Principal Investigator, require unblinding (such as high severity, unexpected events possibly related to the study drug), the pharmacist will request the unblinding process. The pharmacist will consult the treatment assignment list and inform the Investigator of the assigned treatment for the patient through email. The unblinding process will be documented in the electronic medical record, RedCap, and will be officially notified to the Sponsor and the Ethics Committee (CIEI), including the date and the reason for the unblinding. Monitors with access to unblinded records will be able to verify that the dispensing was conducted in accordance with the randomization scheme for quality assurance purposes.

## Data collection and management

### Plans for assessment and collection of outcomes {18a}

All participants in the clinical trial will undergo scheduled evaluations as outlined in the study protocol. To ensure smooth coordination of these activities, a dedicated coordinator from the research center will be responsible for organizing and overseeing the necessary procedures. Additionally, prior to the commencement of the study, all team members will receive comprehensive training to familiarize themselves with the protocols and ensure the accuracy and validity of the results. The administration of scales and evaluations will strictly adhere to the established procedures to maintain consistency and reliability throughout the study.

### Plans to promote participant retention and complete follow-up {18b}

At the outset of enrollment, all participants are thoroughly informed about the significance of adhering to the instructions and recommendations provided by the research team. They are encouraged to raise any questions or concerns they may have and assured of continuous support through regular telephone communication and follow-up visits. In cases where a patient fails to attend scheduled visits, the research team will proactively reach out to their relatives to ascertain the reason for the absence and aid ensure compliance with the planned visits. This proactive approach aims to maintain the continuity and completeness of the study data while prioritizing the well-being and engagement of the participants.

### Data management {19}

A electronic case report form (eCRF) will be completed for each selected and enrolled subject in the study. The eCRF data will be captured using an electronic data capture system (CED), specifically designed by the statistics and epidemiology section of the Research Center at Hospital Universitario San Ignacio in RedCap ®. The investigator will maintain subject files where all subject data will be documented. These files serve as the primary source of data for the study. The research center staff will accurately record all required eCRF data specified in the protocol within the system. All data entered into the eCRF will be supported by corresponding source documentation.

If necessary, the investigator or an authorized member of the investigator's team will make any required corrections to the eCRF. The audit trail, an integral part of the system, will record all modified information, including the date and person responsible for the correction. Periodic reviews of the eCRFs will be conducted by CRO staff to ensure completeness, legibility, and compliance. Access to all source documents pertaining to the study will be granted to verify the accuracy of data entered the eCRF. The Principal Investigator will review the eCRFs for completeness and accuracy, electronically signing and dating them as confirmation of their review.

### Confidentiality {27}

The information generated during this clinical study is deemed confidential and will be utilized solely for the purposes related to the development of Esperanza extract. This confidential information will remain the exclusive property of the sponsors and shall not be disclosed to any third parties without written consent. It is strictly prohibited to use this information for any purpose other than the proper execution of this study. Additionally, the personal data of the participants will be kept confidential and will not be disclosed at any point during or after the study. Proper anonymization measures will be implemented to ensure the protection of participants' privacy.

### Plans for collection, laboratory evaluation and storage of biological specimens for genetic or molecular analysis in this trial/future use {33}

N/a: The collection of all data and biological samples for this study is an integral part of the protocol activities. After completing analysis, samples will be discarded following standardized hospital protocol.

## Statistical methods

### Statistical methods for primary and secondary outcomes {20a}

A descriptive analysis of the study database will be conducted, including measures of central tendency (such as mean, median, and mode) and dispersion (such as standard deviation and range) for quantitative variables, as well as frequencies and counts for categorical/ordinal variables. In cases where data is missing, an active search will be performed in the electronic clinical record system (SAHI®) of the Hospital, clinical laboratory database (LabCore®), and directly from the patient to retrieve the missing information.

No imputation method is expected to be used, and only patients with complete information will be included in the analysis for each specific objective. For exploratory analyses, the normality of each quantitative variable will be assessed to determine the appropriate use of statistical tests. Depending on the distribution of the data, parametric tests such as the Student's t-test and ANOVA may be used for comparisons between groups, while non-parametric tests such as the Kruskal–Wallis and Mann–Whitney test may be used for non-normally distributed data. The Logrank test will be utilized for survival outcomes associated with that objective.

### Interim analyses {21b}

At the end of phase I, an analysis of the safety profile will be conducted to evaluate the adverse events and determine if any additional safety tests or measures need to be implemented in phase II. This analysis will provide valuable insights into the safety and tolerability of the intervention.

Based on the initial results from phase I, the sample size for phase II will be recalculated, taking into consideration the outcomes observed during phase I. This recalculation aims to ensure an appropriate sample size that allows for robust statistical analysis and meaningful conclusions in phase II.

### Methods for additional analyses (e.g. subgroup analyses) {20b}

N/a: No additional analysis is planned.

### Methods in analysis to handle protocol non-adherence and any statistical methods to handle missing data {20c}

In cases where data is missing, an active search will be performed in the electronic clinical record system (SAHI®) of the Hospital, clinical laboratory database (LabCore®), and directly from the patient to retrieve the missing information. No imputation method is expected to be used, and only patients with complete information will be included in the analysis for each specific objective.

### Plans to give access to the full protocol, participant level-data and statistical code {31c}

The protocol and the data generated to support the findings of this study will be made available from the corresponding author upon reasonable request.

## Oversight and monitoring

### Composition of the coordinating center and trial steering committee {5d}

All study personnel are affiliated with the Hospital Universitario San Ignacio and the Pontificia Universidad Javeriana and have received approval from the ethics committee to participate in the study. The study will be conducted following the principles and guidelines of Good Clinical Practice (GCP). A designated coordinator will be responsible for overseeing and coordinating all protocol activities, ensuring compliance with the study procedures. In addition to the coordinator, the research center will have a diverse team of professionals, including bacteriologists, pharmaceutical chemists, supporting doctors, and nurses, who have also received approval from the institutional review board (IRB) to actively contribute to the study.

### Composition of the data monitoring committee, its role and reporting structure {21a}

The data monitoring committee for this protocol consists of the leader of the research center's safety research program at the Hospital Universitario San Ignacio and an external monitor from the Contract Research Organization (CRO). They will conduct regular reviews of the electronic case report forms (eCRFs) of the patients to ensure the integrity, legibility, and acceptability of the data. The committee members will have access to all source documents related to the study to verify the consistency of the data recorded in the eCRFs. This allows for cross-referencing and confirmation of data accuracy.

### Adverse event reporting and harms {22}

The investigator will actively monitor all study subjects for any occurrence of adverse events (AEs), both clinically and through laboratory tests, throughout the study period. Detailed documentation will be made for each AE, including the date of onset, diagnosis (if known) or presenting signs/symptoms, severity, duration (end date, ongoing, intermittent), relationship to the study drug, and actions taken in response. While it is not expected, in the event of pregnancies, comprehensive follow-up will be conducted after obtaining informed consent from the pregnant women and for data collection on both the mother and the newborn. For serious adverse events (SAEs) that are of an intermittent nature, they must demonstrate similarity in terms of their nature and severity. All adverse events will be recorded, whether prompted by specific questions, observed by the center's staff, or self-reported by the subjects themselves.

Monitoring of adverse events will continue until the resolution of each event (if it is expected to resolve) or until the clinical staff determines that it is appropriate within a 4-month period following the onset of the adverse event. Any serious adverse events (SAEs) experienced by study participants and directly related to the therapeutic intervention using the Esperanza extract must be promptly reported. The reporting of adverse events and the grading of their severity will adhere to the NCI CTCAE v5 guidelines.

### Frequency and plans for auditing trial conduct {23}

A comprehensive review of all data will be conducted in accordance with the monitoring plan established for the study to ensure its accuracy and maintain data quality. The CRO monitor will oversee the research center's activities throughout the study duration. Source documents will be reviewed against the corresponding entries in the case report forms, and a quality assurance review will be conducted to ensure that the investigator adheres to the protocol and regulatory requirements. Any discrepancies identified will be cross-checked against the written case report form and corrected in the electronic system. Following data input, both manual and automated logic checks will be implemented to identify potential issues, such as inconsistent study dates, thereby ensuring the integrity of the data.

### Plans for communicating important protocol amendments to relevant parties (e.g. trial participants, ethical committees) {25}

If any amendments are made to the study, they will be submitted for approval to the ethics committee of the San Ignacio University Hospital. Once approved by the ethics committee, the study will seek approval from the national regulatory agency for implementation. Prior to implementation, comprehensive training will be provided to all personnel involved in the study, tailored to their respective roles and responsibilities within the protocol.

The clinical protocol, informed consents, and investigator's manual used in this study have already been approved by both the ethics committee and the national regulatory agency in their clinical study review. The study will be conducted in accordance with the Good Clinical Practice (GCP) guidelines of the International Conference on Harmonization (ICH), as well as the relevant regulations and standards governing the conduct of clinical studies. Additionally, the study will adhere to the ethical principles outlined in the Declaration of Helsinki.

An initial amendment was made in response to a request from the National Regulatory Agency, INVIMA. This amendment received approval during the ordinary session held on May 25, 2023, under Act No. (9/2023), evaluated by the Research and Institutional Ethics Committee of the Faculty of Medicine at Pontificia Universidad Javeriana and Hospital Universitario San Ignacio.

### Dissemination plans {31a}

The findings from this clinical trial are anticipated to be shared through conference presentations and published in peer-reviewed journals.

## Discussion

Despite the wide variety of highly purified antitumor products available, herbal products derived from traditional medicine are increasingly being used as an alternative therapy. These products are used to reduce the side effects and organic toxicity of conventional therapies, stimulate the immune system, and prevent the appearance or recurrence of neoplasms as observed with the positive results from traditional evidence [22] and clinical trials on gastric cancer [23, 24] and hematopoietic diseases [25].

*P. alliacea* has been widely used in traditional medicine for the treatment of leukemia and solid tumors [17, 21, 26]. Moreover, its activity on tumor metabolism [18, 19] makes it a therapeutic candidate for a wide variety of tumors that present alterations in glycolytic metabolism and depend on mitochondria to produce energy [27–29]. Then, the modulating activity of cell metabolism exerted by the anamu extract may be responsible, at least in part, for the traditionally recognized antitumor activity.

The extract developed in the last 15 years, which we call Esperanza, has met all the pharmaceutical requirements, including chemical and biological standardization, as well as preclinical studies of safety and efficacy according to the FDA, US and Colombia guidelines for herbal products [30, 31] to start clinical studies. Safety will be evaluated in this phase I/II clinical trial, but phase III clinical trials will be needed to evaluate efficacy in patients with gastric tumors and acute leukemias.

Acceptance of these therapies by conventional physicians and patients may present an additional challenge in this clinical study, which could affect patient recruitment and retention. To address this challenge, there is a research team made up of hematologists, oncologists, scientists specialized in phytomedicine and immunology, as well as educational programs and continuous follow-up of patients, all of which are integrated during the design and execution of the protocol.

This PA001 clinical study is the first to evaluate the activity of Anamu in a controlled manner, and its objective is to determine the safety profile of the extract of this plant in patients with gastric tumors and acute leukemia, as well as to explore efficacy results that allow it to continue and improve the clinical development of this plant as a phytomedicine for the treatment of neoplasias. It is noteworthy that despite its history of traditional use, the rigorous process of clinical validation is a fundamental requirement for incorporating phytomedicines into the treatment of different diseases based on scientific rationales.

## Trial status

The recruitment of patients will start in July 2023 and conclude by February 2024 at the Centro Javeriano de Oncologia of the Hospital Universitario San Ignacio if the sample size is achieved.

### Supplementary Information

**Additional file 1.** contains the checklist for the Study Protocols (SPIRIT) 2013 recommendations [32].

## Data Availability

The protocol and the data generated to support the findings of this study will be made available from the corresponding author upon reasonable request.

## Abbreviation

n/a: The abbreviations used in this document have been previously defined in the preceding sections
